# The Medical Adviser and General Practitioner Trials

**Published:** 1980

**Authors:** R. P. Bax

**Affiliations:** Medical Adviser, Roussel Laboratories Ltd., Wembley Park, Middlesex


### The Medical Adviser and General Practitioner Trials

**R. P. Bax, M.B., B.S., M.R.C.G.P., Dip.Pharm.Med.**
Medical Adviser, Roussel Laboratories Ltd., Wembley Park, Middlesex

As an ex-general practitioner now working in the pharmaceutical industry, I believe in the maxim 'Better Medicines for Common Illnesses'. But such medicines will not appear without years of hard work and co-operation between practising doctors and their colleagues in the pharmaceutical industry. I have already written on the reasons why I left general practice and entered the pharmaceutical industry.[1] In this article I will discuss the background against which the medical adviser works and argue the case for general practitioner trials.

Several million pounds are spent during the development of a drug and ten million more if the drug is destined to become a major international product. Many thousands of compounds are synthesised but only one or two chosen. The time lag from the first synthesis of a successful drug to marketing now averages about eight years. Soon, with increasing legislation and drug control, the pre-registration time spent accumulating the necessary data for a product licence (permission to market the drug), because of continuing technological advances in methodology, will exceed ten years and much more rigorous testing than before is necessary. This includes detailed animal toxicology, both short and long term, in at least two animal species, teratology and fertility studies, carcinogenicity studies, human and animal pharmacology, pharmacodynamic and pharmacokinetic studies, as well as large scale clinical trials often involving many thousands of patients. Thus the breadth of knowledge available at launch of a new product is often much greater than the data available on existing compounds many of which are prescribed frequently. With new compounds the story may not end there. The Committee on Safety of Medicines may require further data collection on several thousand patients treated with the new drug. Various schemes for detecting rare or unusual side-effects have been described including post-marketing surveillance and monitored release. One scheme, adopted during the early release of buprenorphine, a potent analgesic, has just been reported by A. W. Harcus et al.[2] Their conclusion was that this particular type of monitored release did not further increase the knowledge of the drug in terms of efficacy or side effects. The effort was also very expensive in terms of both human and financial resources. The escalating cost and increasing time that has to be expended before releasing a drug for general use will inhibit the research and development of almost all new drugs. Paradoxically, increasing costs may stifle the emergence of all but a few new novel drugs since the pharmaceutical houses are unlikely to invest large sums of money into high risk ventures, and instead will concentrate even more on the further development of well tried and tested 'me-too' drugs. As many general practitioners will admit, common illnesses are often treated badly, i.e. the common cold, backache, and so on, and what are really needed are simple, safe and better treatments for these conditions! What is especially interesting to me is that one of the major factors affecting the direction of research in the pharmaceutical industry is the size of the potential market for the new medicine. This is largely determined by the incidence of the disease to be treated and by the possible advantages of the new compound to both patient and doctor. In this way the maxim of better medicines for common illnesses that are poorly treated is being fulfilled by the pharmaceutical development of 'me-too' drugs. Sir Derek Dunlop in his excellent review entitled 'The innovation, benefit, drawbacks and control of drugs',[3] thought that 'me-too' drugs should not be unduly denigrated, for just as the modern car has gradually evolved from often apparent minor modifications, so modern pharmaceuticals, many of which evolve the same way, often constitute great improvements on their originals.

The Medicines Act of 1968,[4] which came into force on the 1st September 1971, replaced the voluntary compliance with the Committee on Safety of Drugs, which followed directly from the thalidomide disaster of 1959 and brought under legal control all aspects of the manufacture, sale and supply of medicinal products. Through its several Committees, the Medicines Commission advises the Health Ministers on matters relating to the execution of the Act and is also responsible for policing the Act. The Committee on Safety of Medicines is one of these Committees and deals specifically with the granting of clinical trial certificates (authorisation to perform clinical trials), product licences (authorisation to market a drug) and the monitoring of adverse reactions. To obtain these certificates the pharmaceutical company has to submit satisfactory dossiers, which in the case of a product licence application has to show that the product meets standards of efficacy, safety and quality which are acceptable in the present state of knowledge and experience.

The Code of Practice for the Pharmaceutical Industry is a voluntary code created by the Association of the British Pharmaceutical Industry (ABPI) after consultation with the BMA and DHSS. This code specifically deals with promotion of pharmaceutical products and all member companies of the ABPI are pledged to abide by it. The Medicine Act also concerns itself with this subject, but the ABPI feels that appropriate standards of marketing conduct cannot be defined by statute. The code of practice, which is reproduced in the front of the Data Sheet Compendium, deals with such factors as methods of promotion, printed material, claims, data sheets, gifts, audio visual material and the conduct of medical representatives. Recently there has been criticism that some pharmaceutical companies have been carrying out 'bogus' clinical trials with the main aim of promoting their drug rather than for any scientific reasons. The situation is complex and genuine motives may be easily misunderstood. I do not propose to discuss this in detail except to say that a separate ABPI code of practice dealing exclusively with general practitioner trials is shortly to be published. This should give useful guidelines to both practising physicians and the pharmaceutical industry alike on how ethically to perform general practitioner trials.

I will now discuss some essential differences that I see between general practitioner and hospital trials. General practitioners see and treat many more patients than their hospital colleagues. The average general practitioner sees more than 60 per cent of the patients in his practice list each year whereas only about 20 per cent of this list is seen by hospital doctors.[5] General practitioners treat more than 90 per cent of patients suffering from depressive illness without referral to a consultant psychiatrist. Therefore to test an antidepressant drug for general use without large scale general practitioner trials would be ludicrous. However, general practitioner and hospital studies are essentially different, even the types of patients treated are different! The patients in hospital have already been selected by general practitioners as difficult cases, they will probably have been in psychiatric hospitals before and have in general been treated before and therefore in clinical trial terms are drug contaminated. A washout period is therefore essential. These patients will also have greater social problems than the general practitioner patients and perhaps benefit less from antidepressants and probably more from custodial care. Hospital trials are usually more reliable than general practitioner trials because hospital doctors have more time than busy general practitioners for individual patients. Clinical trials essentially assess change in the status of patients. It is therefore important to obtain reliable base line data against which to assess any change. This is important particularly in psychiatric studies since many of the symptoms of depression such as lethargy, dry mouth, disturbances of sleep and tachycardia, are the same as those recognised as side effects of anti-depressant drugs. Many general practitioner trials are multicentre and suffer from the many problems of multicentre trials. These include different standards of admission and assessment, inconsistent data collection and long duration. Indeed, the general practitioner patients may not even be clinically depressed, but rather exhibit a depressive reaction underlying the only too well known syndrome of chronic inability to cope. Recently it has been found that about 40 per cent of patients do not take the medicines prescribed for them. This figure may be even higher and is particularly high in the case of antibiotics and psychoactive drugs. Tablet counts or using marker substances such as riboflavin that show up in the urine are not usually reliable. One would think that in hospital practice non-compliance is no problem. However, those of us who have had relatives in hospital know that this is not so. Junior hospital staff frequently change appointments, but the general practitioner provides personal, private and continuing care to his patients over many years. This fact together with the general practitioner's interest should provide the basis for good trials. Unlike the hospital consultant, he cannot delegate his work but with the increasing employment of paramedical staff, appointment systems and group practices, the general practitioner has, through a reduced consultation rate, more time to devote to other things including clinical research. Well performed clinical trials are of direct benefit to patients who in the end receive better treatment; to doctors since the results of the trials will aid and direct his prescribing; to the economy since useless therapy is avoided and to the pharmaceutical company since they will discover the full potential role and usefulness of their drugs. Patients in a clinical trial often feel they get better attention than patients not in a clinical trial. Better treatment may, of course, be no treatment[6] and when this is in doubt a placebo group should be included in the trial design. Critical factors in drug evaluation consist of randomised, double blind, prospective studies. In the design, performance and interpretation of clinical trials there are many pitfalls and it is perhaps not remarkable that there has never been a perfect clinical trial. Trials are useful for illustrating marginal effects whether in medicine or agriculture. However, the interpretation of the results in medicine are difficult. Whereas in agriculture a 5 per cent increase in crops will result in many hundreds of tons of extra food, in cancer chemotherapy an improvement of 5 per cent in the 10 year survival rate is difficult to measure and to interpret. In the early clinical trials comparing aspirin with cortisone for the treatment of rheumatoid arthritis, it was reported that cortisone was no better than aspirin because the only parameter monitored was pain and no regard was devoted to any improvement in movement or stiffness. Therefore careful thought has to be devoted to the parameters to be measured.

In this article I have discussed the work of the medical adviser in the pharmaceutical industry and contrasted some of the differences between hospital and general practitioner trials. An interested general practitioner along with an informed medical adviser is a potent and valuable source of increased knowledge of drug therapy which neither can afford to neglect and which will be of undoubted benefit to patients in whose interests both are primarily concerned.

### REFERENCES

1. BAX, R. P. Talking Shop. *World Medicine 1979,* 10th March 1979, **10.**
2. HARCUS, A. W., WARD, A. E. and SMITH, D. W. Methodology of monitored release of a new preparation. *BMJ,* 1979, Vol.2 163–165.
3. DUNLOP, Sir D. The innovation, benefits, drawbacks and control of drugs. *Journ.Roy.Soc.Med.,* 1978, Vol.71 324–330.
4. The Medicines Act 1968. Chapter 67. HMSO London.
5. Royal College of General Practitioners, Office of Population Censuses and Surveys and DHSS. 1974. Morbidity statistics from general practice. Second National Study 1970–71. HMSO London.
6. THOMAS, K. B. The consultation and the therapeutic illusion. *BMJ,* 1978, Vol.1 1327–1328.

